# Supplementary material for: Auto‐inhibition of Mif2/CENP‐C ensures centromere‐dependent kinetochore assembly in budding yeast
Source: EMBO J. 2020 Jun 9;39(14):e102938. doi: 10.15252/embj.2019102938 (PMC7360964; doi:10.15252/embj.2019102938)
Supplement: Supplementary file 1 — Appendix [file EMBJ-39-e102938-s001.pdf]

Appendix

Auto-inhibition of Mif2/CENP-C ensures centromere-dependent kinetochore assembly in budding yeast

Appendix Figure S1 – Mif2swap is synthetic lethal with ctf19Δ 2

Appendix Figure S2 – Mif2swap overexpression is toxic for the cell 2

Appendix Figure S3 – Mif2-Flag constructs are expressed to equal levels in yeast cells 3

Appendix Table S1 – Antibodies used in western blotting 4

Appendix Table S2 – Protein expression and yeast strain generation vectors ... 4

Appendix Table S3 – Yeast strains 6

Appendix Table S4 – Organisms and accession numbers 11

Appendix Figure S1

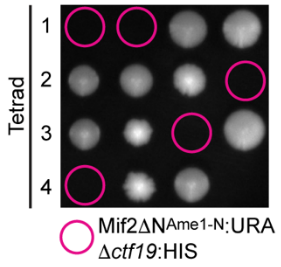

Appendix Figure S1 – Mif2swap is synthetic lethal with ctf19Δ

Tetrad dissection showing inviability of Mif2$^{Ame1\text{-}N}$ in a ctf19Δ background. Inferred genotype of missing spores is indicated.

Appendix Figure S2

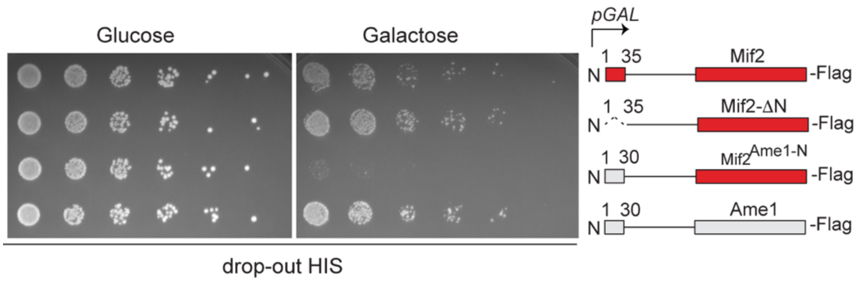

Appendix Figure S2 – Mif2swap overexpression is toxic for the cell

Serial dilution assay comparing overexpression of indicated Mif2 versions with overexpression of full-length Ame1 from a pESC-HIS plasmid. Plates are shown after 3 days of growth at 30°C.

Appendix Figure S3

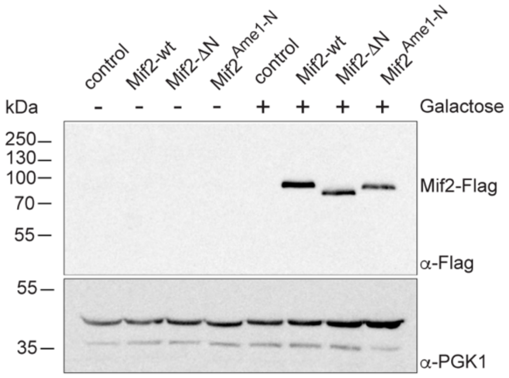

Appendix Figure S3 – Mif2-Flag constructs are expressed to equal levels in yeast cells

Yeast strains harboring Mif2-Flag constructs under a Galactose inducible promotor where grown in Glucose or Galactose containing medium and expression levels analyzed by western blotting. All Mif2-Flag variants have similar expression levels.

Appendix Table S1 – Antibodies used in western blotting

Antibody	Provider	Dilution
Anti-FLAG M2 Monoclonal Antibody-Peroxidase Conjugate	Sigma	1:10000
Phosphoglycerate Kinase Monoclonal Antibody	Invitrogen	1:10000
HA.11 Clone 16B12	Covance	1:2000
Penta-His conjugate HRP	Qiagen	1:1000
c-myc (9E10) monoclonal	BioLegend	1:1000
QIAexpress Strep-tag	Qiagen	1:1000
Peroxidase-conjugated AffiniPure Goat Anti-Mouse IgG	JacksonImmunoResearch	1:5000

Appendix Table S2 – Protein expression and yeast strain generation vectors

Plasmid	Description	Source
Bacterial expression		
pSW698	pST39-Mtw1/Nsl1/Nnf1/6×His-Dsn1	Hornung *et al* 2014
pSW785	pETDuett-6xHis-Nnf1/Mtw1	Hornung *et al* 2014
pSW900	pST39-Okp1/Ame1-6xHis	Hornung *et al* 2014
pKK59	pST39-Mtw1_5A (D25A D81A K82A E84A Y86A) /Nsl1/Nnf1/6×His-Dsn1 (5A mutant)	This study
pKK45	pST39-Mtw1_8A (D25A N32A Y36A E73A D81A K82A E84A Y86A) /Nsl1/ Nnf1_2A (V15A R18A) /6×His-Dsn1 (10A mutant)	This study

pKK37	pST39-Mtw1_3A (E73A N32A Y36A) /Nsl1/Nnf1/6×His-Dsn1 (3A mutant)	This study
pKK62	pGEX-6P1-Mif2_1-41	This study
pSW762	pGEX-6P1-Ame1_1-30	Hornung *et al* 2014
pSW583	pST44-Cse4/H4/H2A/H2B-6xHis-6xFlag	Alwin Köhler Lab
Insect cell expression		
pSW181	Mif2-wt-1xFlag in pFL	Hornung *et al* 2014
pSW1424	Mif2-Δ1-35:Ame1_1-30-1xFlag in pFL	This study
pSW189	Strep-Dsn1, Mtw1, Nnf1, Nsl1 in pFL	Hornung *et al* 2014
pSW926	pBIG1c Mcm16-Mcm22-Ctf3-StrepII-Wip1-Cnn1	Pekgöz Altunkaya *et al* 2016
pSW186	Mif2-ΔNT-FLAG in pFL	Hornung *et al* 2014
pSW1446	Mif2-ΔsigAT-FLAG in pFL	This study
Yeast genetics		
pKK50	pRS306-Mtw1-wt-6xFlag	This study
pKK51	pRS306-Mtw1_3A (E73A N32A Y36A) -6xFlag (3A mutant)	This study
pKK52	pRS306-Mtw1_5A (D25A D81A K82A E84A Y86A) -6xFlag (5A mutant)	This study
pKK53	pRS306-Mtw1_8A (D25A N32A Y36A E73A D81A K82A E84A Y86A) -6xFlag (8A mutant)	This study
pKK14	pRS306-Ame1-wt-3xHA	This study
pKK15	pRS306-Ame1-Δ1-15-3xHA	This study
pKK16	pRS306-Ame1-Δ1-15:Mif2_1-35-3xHA	This study
pKK3	pRS305-Ame1-wt-3xHA	This study
pKK10	pRS305-Ame1-Δ1-15-3xHA	This study
pKK12	pRS305-Ame1-Δ1-15:Mif2_1-35-3xHA	This study
pKK2	pRS306-Mif2-wt-6xFlag	This study
pKK9	pRS306-Mif2-Δ1-35-6xFlag	This study
pKK54	pRS306-Mif2-Δ1-35:Ame1_1-30-6xFlag	This study

pKK80	pGAL-Mif2Δ1-35:Ame1_1-30R10E-1xFlag in pRS306	This study
pSW1427	Mif2-wt-1xFlag in pESC-HIS	This study
pSW1428	Mif2-Δ1-35-1xFlag in pESC-HIS	This study
pSW1429	Mif2-Δ1-35:Ame1_1-30-1xFlag in pESC-HIS	This study
pSW1430	empty pGAL expression cassette in pRS306	This study
pSW1431	pGAL-Mif2-wt-1xFlag in pRS306	This study
pSW1432	pGAL-Mif2-Δ1-35-1xFlag in pRS306	This study
pSW1433	pGAL-Mif2Δ1-35:Ame1_1-30-1xFlag in pRS306	This study
pSW1451	Mif2-ΔsigAT-FLAG in pESC-HIS	This study
pSW1452	Mif2-ΔNΔsigAT-FLAG in pESC-HIS	This study

Appendix Table S3 – Yeast strains

Strain Name	Relevant Genotype	Source
SWy355	Mat a; tor1-1, fpr1::loxP-LEU2-loxP, RPL13A-2xFKBP12::loxP-TRP1-loxP	Haruki *et al* 2008
PSy1	Mat a tor1-1 fpr1::loxP-LEU2-loxP RPL13A-2xFKBP12::loxP Mtw1-FRB::KanMX	This study
PSy5	Mat a tor1-1 fpr1::loxP-LEU2-loxP RPL13A-2xFKBP12::loxP, Mtw1-FRB::KanMX, Mtw1-wt::URA3	This study
PSy6	Mat a tor1-1 fpr1::loxP-LEU2-loxP RPL13A-2xFKBP12::loxP, Mtw1-FRB::KanMX, Mtw1-3A::URA3 (see pKK51)	This study
PSy7	Mat a tor1-1 fpr1::loxP-LEU2-loxP RPL13A-2xFKBP12::loxP, Mtw1-FRB::KanMX, Mtw1-5A::URA3 (see pKK52)	This study

PSy8	Mat a tor1-1 fpr1::loxP-LEU2-loxP RPL13A-2xFKBP12::loxP, Mtw1-FRB::KanMX, Mtw1-8A::URA3 (see pKK53)	This study
KKy160	Mat a/alpha, Ade2/ade2-1, his3Δ200/ his3Δ200, leu2-3,112/leu2-3,112, ura3-52/ura3-52, Lys2/lys2-801, Mtw1/mtw1 Δ::KanMX	This study
KKy167	Mat a/alpha, Ade2/ade2-1, his3Δ200/ his3Δ200, leu2-3,112/leu2-3,112, ura3-52/ura3-52, Lys2/lys2-801, Mtw1/mtw1Δ::KanMX, Mtw1-wt::URA3	This study
KKy168	Mat a/alpha, Ade2/ade2-1, his3Δ200/ his3Δ200, leu2-3,112/leu2-3,112, ura3-52/ura3-52, Lys2/lys2-801, Mtw1/mtw1Δ::KanMX, Mtw1-3A::URA3	This study
Kky169	Mat a/alpha, Ade2/ade2-1, his3Δ200/ his3Δ200, leu2-3,112/leu2-3,112, ura3-52/ura3-52, Lys2/lys2-801, Mtw1/mtw1Δ::KanMX, Mtw1-5A::URA3	This study
KKy170	Mat a/alpha, Ade2/ade2-1, his3Δ200/ his3Δ200, leu2-3,112/leu2-3,112, ura3-52/ura3-52, Lys2/lys2-801, Mtw1/mtw1Δ::KanMX, Mtw1-8A::URA3	This study
SWY536	Mat a; tor1-1, fpr1::loxP-LEU2-loxP, RPL13A-2xFKBP12::loxP-TRP1-loxP, Ame1-FRB::KanMX	Hornung *et al* 2014
KKy38	Mat a; tor1-1, fpr1::loxP-LEU2-loxP, RPL13A-2xFKBP12::loxP-TRP1-loxP, Ame1-FRB::KanMX, Ame1-wt-3xHA::URA3	This study
KKy39	Mat a; tor1-1, fpr1::loxP-LEU2-loxP, RPL13A-2xFKBP12::loxP-TRP1-loxP, Ame1-FRB::KanMX, Ame1-Δ1-15-3xHA::URA3	This study
KKy40	Mat a; tor1-1, fpr1::loxP-LEU2-loxP, RPL13A-2xFKBP12::loxP-TRP1-loxP, Ame1-FRB::KanMX, Ame1-Δ1-15:Mif2_1-35-3xHA::URA3	This study

KKy4	Mat a/alpha, Ade2/ade2-1, his3Δ200/ his3Δ200, leu2-3,112/leu2-3,112, ura3-52/ura3-52, Lys2/lys2-801, Ame1-wt-3xHA::LEU2	This study
KKy10	Mat a/alpha, Ade2/ade2-1, his3Δ200/ his3Δ200, leu2-3,112/leu2-3,112, ura3-52/ura3-52, Lys2/lys2-801, Ame1-Δ1-15:Mif2_1-35-3xHA::LEU2	This study
KKy140	Mat a/alpha, Ade2/ade2-1, his3Δ200/ his3Δ200, leu2-3,112/leu2-3,112, ura3-52/ura3-52, Lys2/lys2-801, Ame1-Δ1-15-3xHA::LEU2	This study
KKy124	Mat a; tor1-1, fpr1::loxP-LEU2-loxP, RPL13A-2xFKBP12::loxP-TRP1-loxP, Ame1-FRB::KanMX, Ame1-wt-3xHA::URA3, Mtw1-GFP::HIS, Spc42-RedStar::NatNT2	This study
KKy125	Mat a; tor1-1, fpr1::loxP-LEU2-loxP, RPL13A-2xFKBP12::loxP-TRP1-loxP, Ame1-FRB::KanMX, Ame1-Δ1-15-3xHA::URA3, Mtw1-GFP::HIS Spc42-RedStar::NatNT2	This study
KKy126	Mat a; tor1-1, fpr1::loxP-LEU2-loxP, RPL13A-2xFKBP12::loxP-TRP1-loxP, Ame1-FRB::KanMX, Ame1-Δ1-15:Mif2_1-35-3xHA::URA3 Mtw1-GFP::HIS Spc42RedStar:: NatNT2	This study
KKy130	Mat a; tor1-1, fpr1::loxP-LEU2-loxP, RPL13A-2xFKBP12::loxP-TRP1-loxP, Ame1-FRB::KanMX, Mtw1-GFP::HIS, Spc42-RedStar::NatNT2	This study
KKy51	Mat a; tor1-1, fpr1::loxP-LEU2-loxP, RPL13A-2xFKBP12::loxP-TRP1-loxP, Mif2-FRB::KanMX	This study
KKy102	Mat a; tor1-1, fpr1::loxP-LEU2-loxP, RPL13A-2xFKBP12::loxP-TRP1-loxP, Mif2-FRB::KanMX, Mif2-wt-6xFlag::URA3	This study

KKy194	Mat a; tor1-1, fpr1::loxP-LEU2-loxP, RPL13A-2xFKBP12::loxP-TRP1-loxP, Mif2-FRB::KanMX, Mif2-wt-6xFlag::URA3 ctf19Δ:: NatNT2	This study
KKy120	Mat a; tor1-1, fpr1::loxP-LEU2-loxP, RPL13A-2xFKBP12::loxP-TRP1-loxP, Mif2-FRB::KanMX, Mif2-Δ1-35-6xFlag::URA3	This study
KKy195	Mat a; tor1-1, fpr1::loxP-LEU2-loxP, RPL13A-2xFKBP12::loxP-TRP1-loxP, Mif2-FRB::KanMX Mif2-Δ1-35-6xFlag::URA3, ctf19 Δ:: NatNT2	This study
KKy157	Mat a; tor1-1, fpr1::loxP-LEU2-loxP, RPL13A-2xFKBP12::loxP-TRP1-loxP, Mif2-FRB::KanMX Mif2-Δ1-35:1-30Ame1-6xFlag::URA3	This study
KKy139	Mat a/alpha, Lys2/lys2-801, ura3-52/ura3-52, his3Δ200/ his3Δ200, leu2-3,112/leu2-3,112, Mif2-Δ1-35:1-15Ame1-6xFlag::URA3, ctf19Δ::HIS3MX6	This study
KKy196	Mat a; lys2-801, ura3-52, leu2-3, his3Δ200, Mif2-wt-6xFlag::URA, Mtw1-13xMyc::His3MX6	This study
KKy200	Mat a; ura3-52, leu2-3, his3Δ200, Mtw1-13xMyc::His3MX6	This study
KKy204	Mat a; lys2-801, ura3-52, leu2-3, his3Δ200, Mif2-Δ1-35-6xFlag::URA3, Mtw1-13xMyc::His3MX6	This study
KKy220	Mat a; ade2-1, ura3-52, leu2-3, his3Δ200, Mif2-Δ1-35:1-30Ame1-6xFlag::URA3, Mtw1-13xMyc::His3MX6	This study
PSy10	Mat a; tor1-1, fpr1::loxP-LEU2-loxP, RPL13A-2xFKBP12::loxP-TRP1-loxP, Mif2-FRB::KanMX, Mif2Δ1-35:1-30Ame1-6xFlag::URA3, ctf19 Δ::NatNT2	This study
SWY2292	Mat a, leu2-3,112, his3Δ200, ade2-1, ura3-52, (pESC-HIS:Mif2-wt-1xFlag)	This study

SWY2293	Mat a, leu2-3,112, his3Δ200, ade2-1, ura3-52, (pESC-HIS:Mif2-Δ1-35-1xFlag)	This study
SWY2294	Mat a, leu2-3,112, his3Δ200, ade2-1, ura3-52, (pESC-HIS:Mif2Δ1-35:1-30Ame1-1xFlag)	This study
SWY2308	Mat alpha, leu2-3,112, his3Δ200, lys2-801, ura3-52::pGAL-Mif2-wt-Flag::URA3	This study
SWY2309	Mat alpha, leu2-3,112, his3Δ200, lys2-801, ura3-52::pGAL-Mif2-Δ1-35-Flag::URA3	This study
SWY2310	Mat alpha, leu2-3,112, his3Δ200, lys2-801, ura3-52::pGAL-Mif2-Δ1-35:1-30Ame1-Flag::URA3	This study
SWY2311	MAT alpha, leu2-3,112, his3Δ200, lys2-801, ura3-52::pGAL::URA3	This study
SWY2320	Mat alpha, promCu-LacI-GFP::HIS3, leu2-2,112::lacO::LEU2, ura3-52:::pGAL-empty::URA3	This study
SWY2321	Mat alpha, promCu-LacI-GFP::HIS3, leu2-2,112::lacO::LEU2, ura3-52:::pGALMif2-wt-1xFlag::URA3	This study
SWY2322	Mat alpha, promCu-LacI-GFP::HIS3, leu2-2,112::lacO::LEU2, ura3-52::pGAL-Mif2-Δ1-35-1xFlag::URA3	This study
SWY2323	Mat alpha, promCu-LacI-GFP::HIS3, leu2-2,112::lacO::LEU2, ura3-52:::pGAL-Mif2Δ1-35:1-30Ame1-1xFlag::URA3	This study
SWY2324	Mat a, leu2-3,112, lys2-801, Mtw1-GFP::KanMX, Spc42-RFP::HIS3, ura3-52::pGAL-empty::URA3	This study
SWY2325	Mat a, leu2-3,112, lys2-801, Mtw1-GFP::KanMX, Spc42-RFP::HIS3, ura3-52::pGAL-Mif2-wt-1xFlag::URA3	This study
SWY2326	Mat a, leu2-3,112, lys2-801, Mtw1-GFP::KanMX, Spc42-RFP::HIS3, ura3-52::pGAL-Mif2-Δ1-35-1xFlag::URA3	This study

SWY2327	Mat a, leu2-3,112, lys2-801, Mtw1-GFP::KanMX, Spc42-RFP::HIS3, ura3-52::pGAL-Mif2-Δ1-35:1-30Ame1-1xFlag::URA3	This study
SWY2331	Mat a, leu2-3,112, lys2-801, Mtw1-GFP::KanMX, Spc42-RFP::HIS3, ura3-52::pGAL-empty::URA3, mad1Δ::NatNT2	This study
SWY2332	Mat a, leu2-3,112, lys2-801, Mtw1-GFP::KanMX, Spc42-RFP::HIS3, ura3-52::pGAL-Mif2-wt-1xFlag::URA3, mad1Δ::NatNT2	This study
SWY2333	Mat a, leu2-3,112, lys2-801, Mtw1-GFP::KanMX, Spc42-RFP::HIS3, ura3-52::pGAL-Mif2-Δ1-35-1xFlag::URA3, mad1Δ::NatNT2	This study
SWY2334	Mat a, leu2-3,112, lys2-801, Mtw1-GFP::KanMX, Spc42-RFP::HIS3, ura3-52::pGAL-Mif2Δ1-35:Ame_1-30-1xFlag::URA3, mad1Δ::NatNT2	This study
SWY2352	MATa, leu2-3,112, his3Δ200, ade2-1, ura3-52, (pESC-HIS:Mif2-ΔsigAT-1xFlag)	This study
SWY2353	MATa, leu2-3,112, his3Δ200, ade2-1, ura3-52, (pESC-HIS:Mif2-ΔNΔsigAT-1xFlag)	This study

Appendix Table S4 – Organisms and accession numbers

Mif2	
Saccharomyces cerevisiae	CAA81927.1
Candida albicans	AOW30210.1
Hansenicaspora osmophila	OEJ86420.1
Candida glabrata	KTB23823.1
Kluyveromyces marxianus	BAP70857.1
Ame1	

Saccharomyces cerevisiae	KZV13296.1
Candida glabrata	KTB16991.1
Kluyveromyces lactis	XP_452444.1
Saccharomyces eubayanus	XP_018223240.1
Zygosaccharomyces parabailii	AQZ12809.1
