# Supplementary material for: Auto‐inhibition of Mif2/CENP‐C ensures centromere‐dependent kinetochore assembly in budding yeast
Source: EMBO J. 2020 Jun 9;39(14):e102938. doi: 10.15252/embj.2019102938 (PMC7360964; doi:10.15252/embj.2019102938)
Supplement: Supplementary file 2 — Expanded View Figures PDF [file EMBJ-39-e102938-s002.pdf]

Expanded View Figures

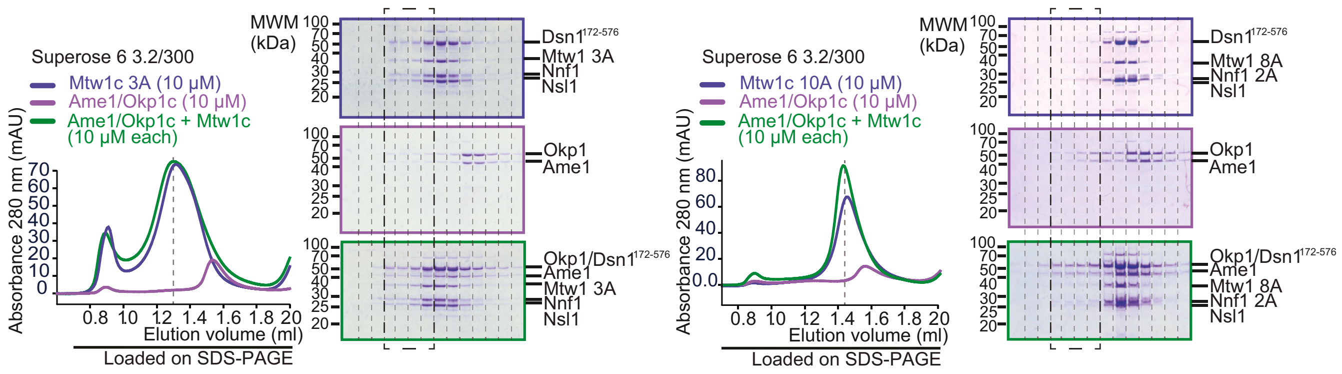

Figure EV1. Mtw1c interface I and I+II mutants abolish AOc binding.

Analytical SEC runs and accompanying SDS–PAGE of additional Mtw1c mutants. Left panel shows Mtw1-3A mutant (interface II mutation), and right panel shows mutant combining Mtw1 mutation 8A (interface I+II) and 2A mutation in Nnf1. Dashed boxes highlighting the corresponding fractions were included to improve comparability.

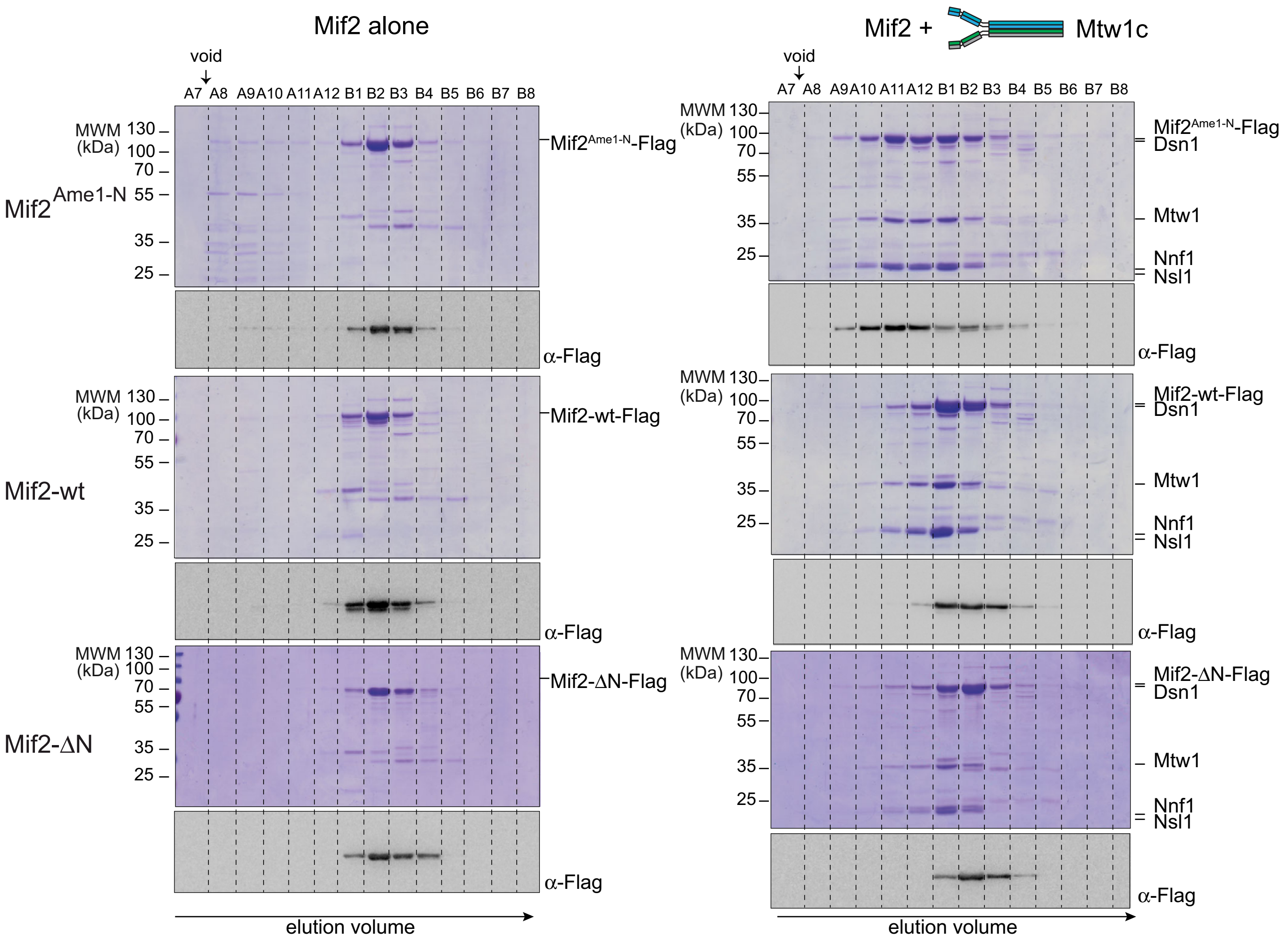

Figure EV2.

© 2020 The Authors

Figure EV2. The Mif2 swap mutant binds more efficiently to Mtw1c.

Coomassie-stained SDS gel and Western blotting of elution fractions from an SEC experiment of Mif2-wt, Mif2$^{Ame1\text{-}N}$ swap, or Mif2-ΔN in the absence (left panel) or presence (right panel) of Mtw1c.

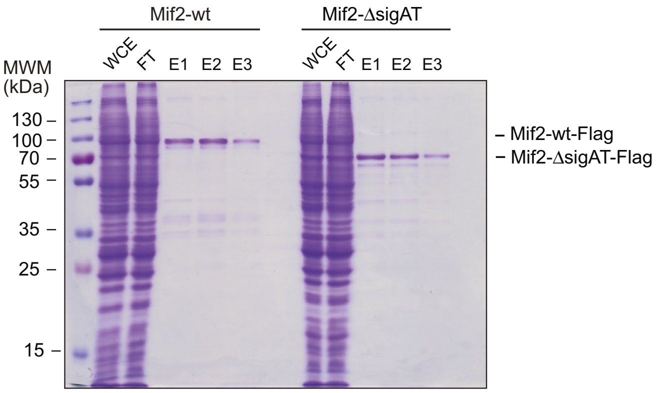

Figure EV3. Mif2-wt and Mif2-ΔsigAT purification from insect cells.

One-step purification of Flag-tagged Mif2-wt or Mif2-ΔsigAT from insect cells and visualization on a Coomassie-stained gel. One-step purification of Flag-tagged Mif2-wt or Mif2-ΔsigAT from insect cells and visualization on a Coomassie-stained gel.

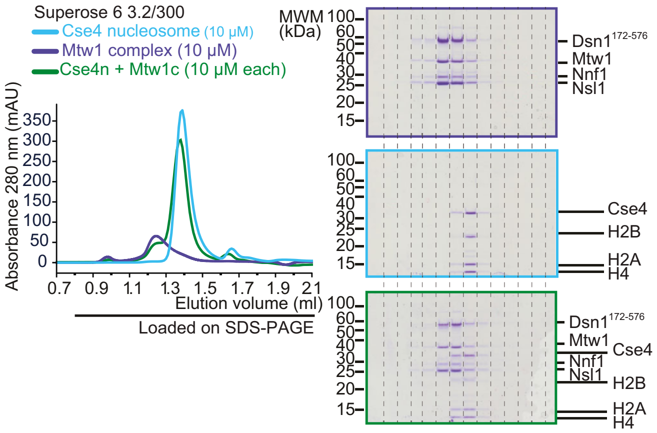

Figure EV4. Cse4 nucleosomes do not directly interact with the Mtw1c.

Analytical SEC and accompanying SDS–PAGE analyzing interaction between Mtw1c (dark blue) and Cse4 mono-nucleosomes (light blue). No appreciable binding can be detected under these conditions (green).

© 2020 The Authors

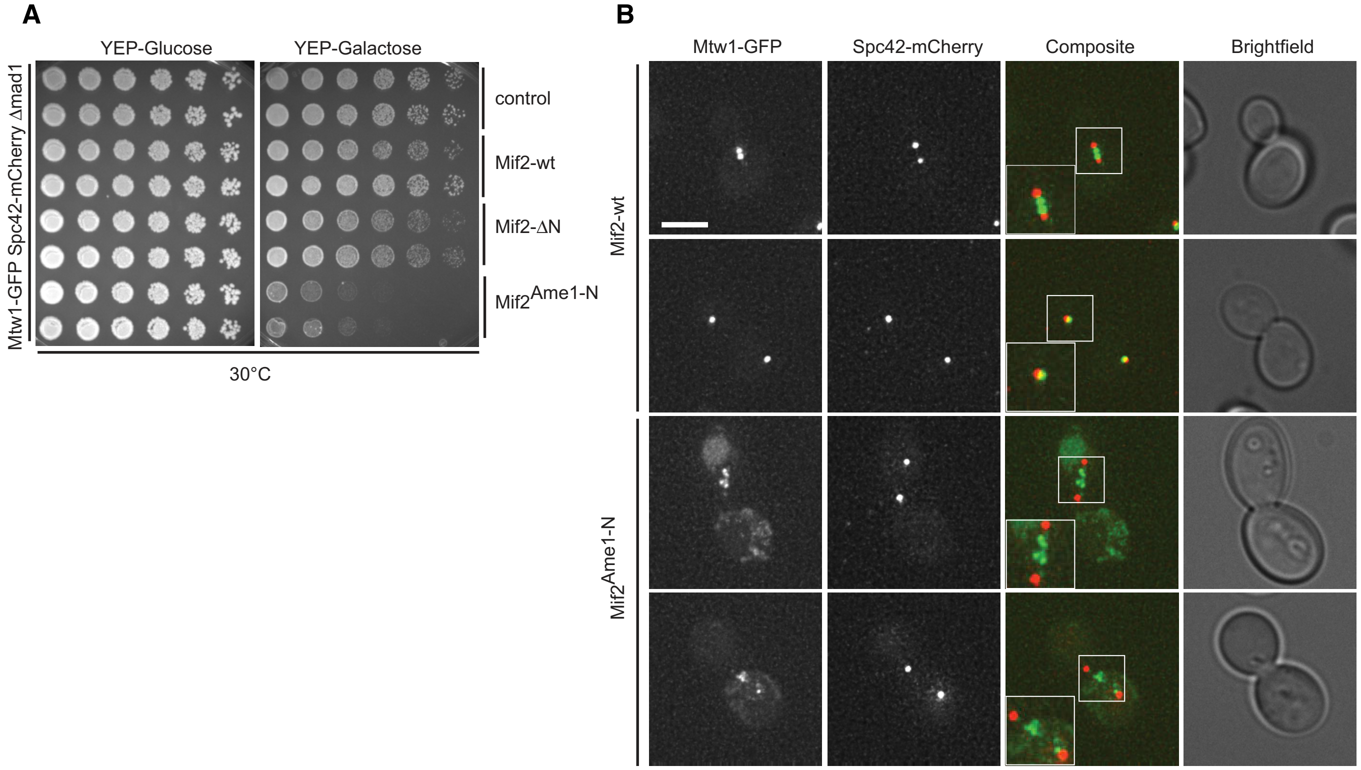

Figure EV5. Auto-inhibition of Mif2 is crucial for cell viability and Mtw1c localization.

A Serial dilution assay of integrated pGAL-Mif2 constructs in a mitotic checkpoint-deficient *mad1*Δ strain. Plates are shown after 3 days of growth at 30°C.

B Live-cell fluorescence microscopy of kinetochores (Mtw1-GFP) and spindle pole bodies (Spc42-mCherry) in cells overexpressing Mif2-wt or $Mif2^{Ame1\text{-}N}$ swap. Boxes show 1.5× enlargement of the indicated area. Scale bar represents 3 μm. Bright-field images show the morphology of the corresponding yeast cell. Only large-budded cells were included in the analysis.

 © 2020 The Authors
